# The Impact of Neck Cooling on Serum Oxidant/Antioxidant Status and HSP70 Levels during High-Intensity Cycling

**DOI:** 10.3390/life13112178

**Published:** 2023-11-08

**Authors:** Kyung-Su Choi, Hee-Tae Roh, Su-Youn Cho

**Affiliations:** 1 Exercise Physiology Laboratory, Department of Physical Education, Yonsei University, Seoul 03722, Republic of Korea; 2 Division of Sports Science, College of Arts and Sports, Sun Moon University, Asan 31460, Republic of Korea

**Keywords:** cycling performance, cooling strategies, oxidative stress, antioxidant enzyme, HSP70, juvenile athletes

## Abstract

Numerous studies have been conducted in an attempt to discover cooling strategies that can be effective in improving exercise performance. However, the mechanism by which neck cooling relieves exercise-induced physiological stress and the optimal cooling temperature are unclear. This study aimed to investigate the effects of neck cooling at different temperatures during high-intensity cycling on body temperature, physiological variables, oxidant/antioxidant status, heat shock protein (HSP) 70 levels, and exercise performance in adolescent athletes. Seven well-trained male adolescent cyclists (age, 17.00 ± 0.76 years; athletic career, 3.86 ± 0.90 years) participated in three exercise trials involving three cooling regimens: control (CON), low-temperature (7 °C) neck cooling (LNC), and mixed-temperature (14 + 20 °C) neck cooling (MNC). The experimental condition used a cross-over design to minimize adaption to the repetitive cycling trials. Cycling consisted of a 20 km warm-up session and a two 2 km race session. Neck cooling at different temperatures was administered for 20 min during each rest period: after the warm-up, after the first 2 km race, and after the second 2 km race. Blood samples were taken to assess serum malondialdehyde (MDA), superoxide dismutase (SOD), and HSP70 levels. In addition, tympanic temperature (Tty), thermal sensation (TS), heart rate (HR), and the saturation of percutaneous oxygen (SpO_2_) were measured before, immediately after, and 24 h after exercise. As a measure of cycling performance, the race record and speed were measured in the first and second 2 km races. In all trials, Tty, TS, HR, MDA, SOD, and HSP70 levels significantly increased (*p* < 0.05), and SpO_2_ levels significantly decreased (*p* < 0.05). TS significantly decreased 24 h after exercise compared to that immediately after exercise in the LNC and MNC trials (*p* < 0.05). Serum HSP70 levels were significantly higher 24 h after exercise (0.87 ± 0.10 ng/mL) than immediately after exercise (0.79 ± 0.04 ng/mL) in the CON trial (*p* < 0.05). Twenty-four hours after exercise, the CON (0.87 ± 0.10 ng/mL) trial showed significantly higher serum HSP70 levels than the LNC (0.73 ± 0.01 ng/mL) trial (*p* < 0.05). There was no significant difference in cycling race record or speed between the trials (*p* > 0.05). Our findings suggest that neck cooling can induce a positive effect on thermal perception during recovery after cycling and that neck cooling at a relatively low temperature may be more effective in reducing exercise-induced HSP70 expression.

## 1. Introduction

Humans are homeotherms; they maintain a constant body temperature through thermoregulatory mechanisms such as heat generation and heat loss. The regulation of body temperature is important as it can have various effects on cellular structure and metabolic pathways [1,2]. The body generates internal heat through normal metabolic processes. However, the amount of metabolic heat generated during rest or sleep is low. During exercise, a large amount of heat is generated, and a decrease in the heat-buffering functions generated by high-intensity exercise can cause excessive heat stress on the body [1,3]. During high-intensity exercise, it is difficult to maintain a normal body temperature and body fluid homeostasis. This poses an increased risk of hyperthermia and heat damage to the body. In particular, thermal damage associated with accelerated muscle fatigue, cardiovascular dysfunction, and central nervous system dysfunction has been suggested as a major cause of decreased exercise performance in athletes [4]. In other words, endurance exercise induces excessive body fluid losses and consequent electrolyte imbalances, and increases the possibility of hyperthermia owing to a rapid rise in body temperature. By activating neurotransmitters, hyperthermia fatigues the central nervous system. Consequently, hyperthermia limits muscle–nerve transmission, inhibits motor unit activity, and causes muscle fatigue [5,6].

Accordingly, in previous studies, various cooling strategies have been proposed to artificially reduce the increase in body temperature during exercise; these can be divided into internal and external cooling [5,7]. Internal cooling aims to lower the core body temperature and relieve thermal strain by creating a heat sink (via cold fluid or ice ingestion). It has been suggested that external cooling strategies—such as cooling garments, cold-water immersion (CWI), and fanning—can reduce thermal strain by increasing the core-to-skin temperature gradient and improving thermal perception [5,8,9]. It has been reported that implementing these cooling strategies can be effective not only in suppressing the increase in body temperature caused by exercise but also in relieving various stresses on the body, such as oxidative stress (OS), inflammation, and muscle damage [10,11]. Pawłowska et al. [10] reported that CWI alleviated the exercise-induced inflammatory response after 30 min of cycling at a 70% maximum heart rate (HR) intensity in healthy adult males. Lindsay et al. [11] reported that, after mixed martial arts training, CWI could reduce the levels of urinary neopterin and total neopterin—which are OS markers—and alleviate delayed-onset muscle soreness. However, CWI is difficult to implement in field-based settings; additionally, it has the potential disadvantage of lowering muscle temperature below the optimal physiological state [5].

Cooling garments such as neck collars, ice towels, and cooling packs have been reported to have the following advantages: they are easy to apply and implement, can be provided in various shapes and sizes to suit the type of sport, and can be set to a specific cooling temperature [5,7]. Accordingly, several studies have reported the positive effects of neck collars [5,7,8,9,10,11,12,13]. Tyler et al. [12] reported that a cooling collar can significantly increase a 15 min running exercise performance. Gordon et al. [13] reported a significant decrease in rectal temperature and sweat rate in 10 male athletes after a 45 min submaximal run when using neck cooling (rating of perceived exertion (RPE), 15). However, few studies have examined the effects of neck cooling interventions on OS, changes in antioxidant enzyme activity caused by high-intensity exercise, and heat shock proteins (HSPs) that reflect intracellular thermal stress. In addition, the optimal cooling temperature that can relieve stress in the body remains unclear. In particular, HSP70 has been reported to reflect the homeostatic imbalance caused by stressful stimuli such as exercise-induced OS, inflammatory response, and muscle damage, as well as intracellular thermal stress [14]. Therefore, assessing HSP70 level changes following neck cooling interventions at different temperatures will contribute to determining the usefulness of neck cooling in sports fields. Accordingly, this study aimed to verify the effects of neck cooling at different temperatures on body temperature, physiological variables, oxidant/antioxidant status, HSP70 levels, and cycling performance during high-intensity cycling. We hypothesized that neck cooling can reduce body temperature and physiological stress during high-intensity cycling and have a positive effect on cycling performance. In addition, we provide empirical evidence to demonstrate the validity and limitations of neck cooling temperature differences.

## 2. Methods

### 2.1. Participants

Seven healthy Korean male adolescent cyclists (athletic career, 3.86 ± 0.90 years) who fully understood the significance of the experiment and voluntarily expressed their intention to participate were included. The number of participants was determined based on effect size (ES) = 0.40, α value = 0.05, and desired statistical power (1 − β) = 0.80 using G*Power software (version 3.1.9.7; Heinrich-Heine-University, Düsseldorf, Germany). A total of six participants were calculated to be needed. Seven participants were selected after considering dropouts, although none occurred. All participants and their guardians understood the benefits and risks of participating in the study and read and signed a consent form stating that participation could be stopped at any time during the experiment and that there would be no disadvantage to withdrawing participation. The study was conducted in accordance with the guidelines of the Declaration of Helsinki and approved by the Ethics Committee of Yonsei University (IRB No.: HR-1384-05; date of approval: 31 August 2022). The physical characteristics of the participants are presented in Table 1.

### 2.2. Baseline Assessment

Height, body composition, and VO_2_max were measured at baseline. Height and body composition were measured using a stadiometer (SECA213; SECA, Hamburg, Germany) and a bioimpedance analysis (BIA) device (Inbody770; Biospace, Seoul, Republic of Korea), respectively. The participants wore short sleeves and pants and removed all metal prior to body composition measurements. VO_2_max was estimated using Cooper’s 1.5-mile maximal running test [15] as per the following formula: VO_2_max (mL/kg/min) = 91.736 − (0.1656 × body mass (kg)) − (2.767 × 1.5 mile run time (min)).

### 2.3. Cycling and Neck Cooling Interventions

**Figure 1:**
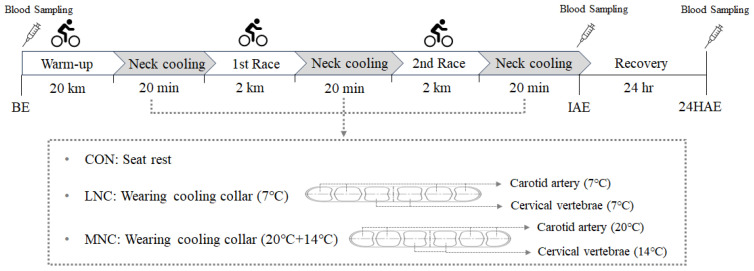
Experimental design. BE, before exercise; IAE, immediately after exercise; 24HAE, 24 h after exercise; CON, control; LNC, low-temperature neck cooling; MNC, mixed-temperature neck cooling.

All participants were student athletes on a cycling team belonging to the same high school. They underwent camp training during the experiment period to control diet, lifestyle patterns, and training status that could affect the study variables. The seven selected participants participated in a total of three different neck cooling interventions during cycling: control (CON), low-temperature (7 °C) neck cooling (LNC), and mixed-temperature (14 + 20 °C) neck cooling (MNC). A rest period of at least one week was provided between each trial. Cycling was conducted in an indoor cycling stadium (mean temperature, 19.19 ± 2.02 °C; mean wind speed, 1.44 ± 0.73 m/s; mean relative humidity, 37.68 ± 5.93%) and consisted of a warm-up session and two races. The warm-up consisted of 20 min cycling on a 333 m track, and the first and second races were conducted using a time-to-distance method of 2 km (333 m × 6 laps) each. Neck cooling was performed for 20 min each after warm-up, after the first race, and after the second race using a neck collar (IceTube; Coolmedics Korea, Hanam, Gyeonggi, Republic of Korea) meant to cool the carotid artery and cervical vertebrae. In the CON trial, the participants rested in a chair without any cooling intervention. On the other hand, neck cooling was mediated at 7 °C for the LNC trial at a mixed temperature of 14 °C and 20 °C for the MNC trial. The experimental condition used a cross-over design to minimize adaption to the repetitive cycling trials as follows: (1) participant numbers ①–④: CON → LNC → MNC, and (2) participant numbers ⑤–⑦: LNC → MNC → CON. The experimental design is illustrated in Figure 1.

### 2.4. Thermoregulatory Responses, Physiological Variables, and Performance Measurement

The thermoregulatory responses were assessed by measuring tympanic temperature (Tty) and thermal sensation (TS) using infrared ear thermometry (Thermoscan IRT 4520; Braun, Kronberg, Germany), and the ASHRAE sensation scale [16], respectively. Physiological variables, HR, and percutaneous oxygen saturation (SpO_2_) were measured using an HR monitor (Garmin 010-12883-00 HRM; Garmin, Olathe, KS, USA) and a pulse oximeter (8500A; Nonin Medical, Plymouth, MN, USA), respectively. Cycling performance was measured using a performance sensor (speed and cadence sensor2; Garmin, Olathe, KS, USA) and PerfPRO software 5.82.06 (PerfPRO Analyzer 2020; PerfPRO Studio, Rockford, MI, USA).

### 2.5. Blood Sampling and Analysis

Blood (5 mL) was collected using a 21-gauge needle and a serum separator tube (Becton Dickinson, Franklin Lakes, NJ, USA) before, immediately after, and 24 h after exercise. The collected blood was centrifuged at 3000 rpm for 10 min, and the separated serum was stored at −80 °C until analysis. Serum malondialdehyde (MDA) (#STA-832; Cell Biolabs, San Diego, CA, USA), SOD (#706002; Chemical, Ann Arbor, MI, USA), and HSP70 (#BMS2087; Thermo Fisher Scientific, Fremont, CA, USA) levels were analyzed using commercially available enzyme-linked immunosorbent analysis kits. All procedures were performed according to the manufacturers’ recommendations.

### 2.6. Statistical Analyses

Statistical analyses were performed using SPSS (version 26.0; IBM Corp., Armonk, NY, USA). Data are expressed as mean ± standard deviation (SD) for all dependent variables. Two-way repeated-measures analysis of variance (ANOVA) was conducted to examine the difference between the time (before, immediately after, and 24 h after exercise) and trials (CON, LNC, and MNC) of each dependent variable. All statistical significance levels (α) were set at 0.05.

## 3. Results

### 3.1. Changes in Thermoregulatory Responses

**Figure 2:**
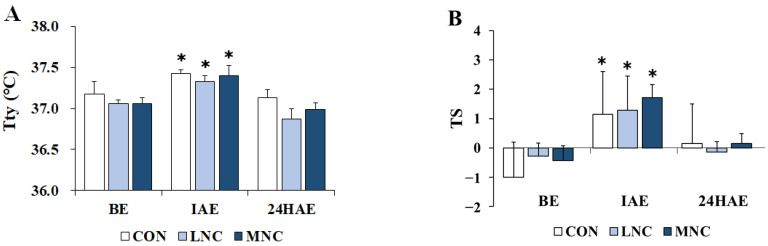
Changes in thermoregulatory responses following neck cooling interventions at different temperatures during cycling. The values are expressed as mean ± SD. (**A**) Tty, tympanic temperature; (**B**) TS, thermal sensation; BE, before exercise; IAE, immediately after exercise; 24HAE, 24 h after exercise; CON, control; LNC, low-temperature neck cooling; MNC, mixed-temperature neck cooling. * Significantly different versus before exercise and 24 h after exercise (*p* < 0.05).

The changes in thermoregulatory responses following the three neck cooling interventions during cycling are shown in Figure 2. The two-way repeated-measures ANOVA conducted on Tty (F = 88.986, *p* < 0.001) and TS (F = 34.357, *p* < 0.001) showed significant differences in time. In all trials, Tty significantly increased immediately after exercise (CON, 37.43 ± 0.05; LNC, 37.33 ± 0.07; MNC, 37.40 ± 0.12 °C, *p* < 0.05) and then significantly decreased 24 h after exercise (CON, 37.13 ± 0.10; LNC, 36.87 ± 0.13; MNC, 36.99 ± 0.08 °C, *p* < 0.05). Meanwhile, TS significantly increased immediately after exercise in all trials (CON, 1.14 ± 1.46; LNC, 1.29 ± 1.16; MNC, 1.71 ± 0.45 score, *p* < 0.05), and then significantly decreased 24 h after exercise in the LNC (−0.14 ± 0.35 score, *p* < 0.05) and MNC (0.14 ± 0.35 score, *p* < 0.05) trials, except in the CON trial.

### 3.2. Changes in Physiological Variables

**Figure 3:**
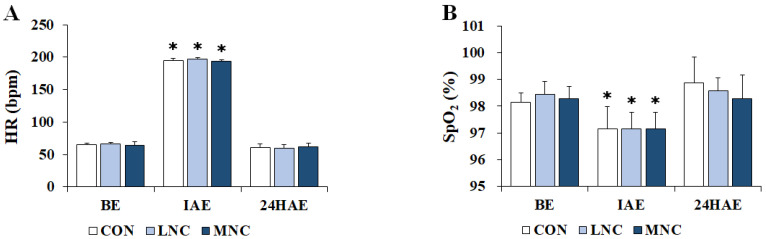
Changes in physiological variables following neck cooling interventions at different temperatures during cycling. The values are expressed as mean ± SD. (**A**) HR, heart rate; (**B**) SpO_2_, saturation of percutaneous oxygen; BE, before exercise; IAE, immediately after exercise; 24HAE, 24 h after exercise; CON, control; LNC, low-temperature neck cooling; MNC, mixed-temperature neck cooling. * Significantly different versus before exercise and 24 h after exercise (*p* < 0.05).

The changes in physiological variables following the three neck cooling interventions during cycling are shown in Figure 3. The two-way repeated-measures ANOVA conducted on HR (F = 6182.614, *p* < 0.001) and SpO_2_ (F = 24.923, *p* < 0.001) showed significant differences in time. In all trials, HR significantly increased immediately after exercise (CON, 194.29 ± 3.49; LNC, 197.57 ± 1.40; MNC, 194.14 ± 1.46 bpm, *p* < 0.05) and then significantly decreased 24 h after exercise (CON, 60.86 ± 4.88; LNC, 59.86 ± 5.46; MNC, 61.43 ± 5.78 bpm, *p* < 0.05). In contrast, SpO_2_ significantly decreased immediately after exercise in all trials (CON, 97.14 ± 0.83; LNC, 97.14 ± 0.64; MNC, 97.14 ± 0.64%, *p* < 0.05), and then significantly increased 24 h after exercise (CON, 98.86 ± 0.99; LNC, 98.57 ± 0.49; MNC, 98.29 ± 0.88%, *p* < 0.05).

### 3.3. Changes in Serum Oxidant/Antioxidant Status

**Figure 4:**
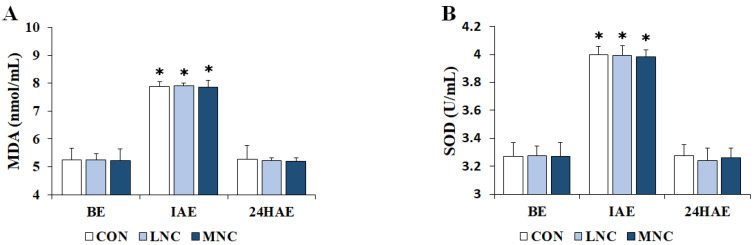
Changes in serum oxidant/antioxidant status following neck cooling interventions at different temperatures during cycling. The values are expressed as mean ± SD. (**A**) MDA, malondialdehyde; (**B**) SOD, superoxide dismutase; BE, before exercise; IAE, immediately after exercise; 24HAE, 24 h after exercise; CON, control; LNC, low-temperature neck cooling; MNC, mixed-temperature neck cooling. * Significantly different versus before exercise and 24 h after exercise (*p* < 0.05).

The changes in serum oxidant/antioxidant status following the three neck cooling interventions during cycling are shown in Figure 4. The two-way repeated-measures ANOVA conducted on MDA (F = 20,716.985, *p* < 0.001) and SOD (F = 10,985.463, *p* < 0.001) showed significant differences in time. Serum MDA levels significantly increased immediately after exercise in all trials (CON, 7.87 ± 0.04; LNC, 7.90 ± 0.02; MNC, 7.86 ± 0.05 nmol/ML, *p* < 0.05), and then significantly decreased 24 h after exercise (CON, 5.27 ± 0.10; LNC, 5.22 ± 0.02; MNC, 5.19 ± 0.02 nmol/mL, *p* < 0.05). In addition, similar to MDA, serum SOD activities significantly increased immediately after exercise in all trials (CON, 4.00 ± 0.02; LNC, 3.99 ± 0.02; MNC, 3.98 ± 0.02 U/mL, *p* < 0.05), and then significantly decreased 24 h after exercise (CON, 3.28 ± 0.03; LNC, 3.24 ± 0.03; MNC, 3.26 ± 0.02 U/mL, *p* < 0.05). However, there was no significant difference between the trials (*p* > 0.05).

### 3.4. Changes in Serum HSP70 Levels

**Figure 5:**
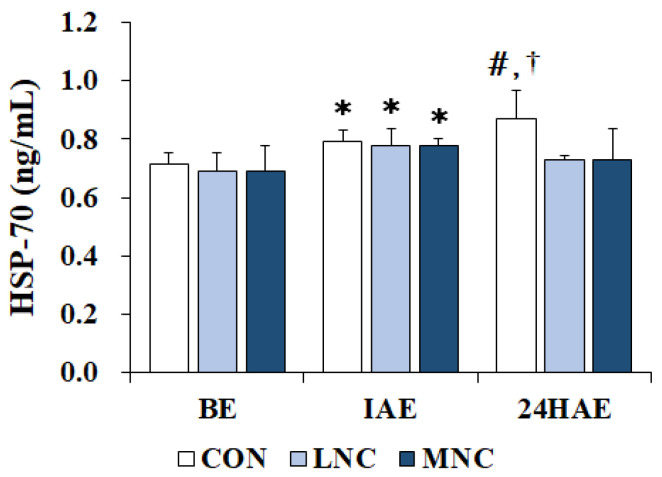
Changes in serum HSP70 levels following neck cooling at different temperatures during cycling. The values are expressed as mean ± SD. HSP70, heat shock protein70; BE, before exercise; IAE, immediately after exercise; 24HAE, 24 h after exercise; CON, control; LNC, low-temperature neck cooling; MNC, mixed-temperature neck cooling. * Significantly different versus before exercise (*p* < 0.05). # Significantly different versus immediately after exercise (*p* < 0.05). † Significant difference with LNC and MNC trials (*p* < 0.05).

The changes in serum HSP70 levels following the three neck cooling interventions during cycling are shown in Figure 5. The two-way repeated-measures ANOVA conducted on HSP70 showed significant differences in time (F = 29.675, *p* < 0.001) and trial (F = 14.850, *p* = 0.001). Serum HSP70 levels significantly increased immediately after exercise in all trials (CON, 0.79 ± 0.04; LNC, 0.78 ± 0.05; MNC, 0.78 ± 0.02 ng/mL, *p* < 0.05). The CON trial showed significantly higher HSP70 levels 24 h after exercise (0.87 ± 0.10 ng/mL) compared to those immediately after exercise (*p* < 0.05). Additionally, 24 h after exercise, the CON trial (0.87 ± 0.10 ng/mL) showed significantly higher HSP70 levels than the LNC trial (0.73 ± 0.01 ng/mL, *p* < 0.05).

### 3.5. Changes in Cycling Race Record and Speed

**Figure 6:**
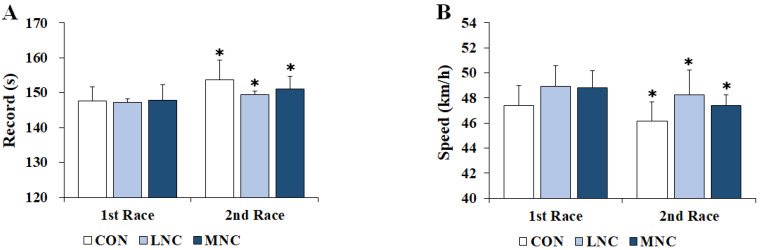
Changes in cycling race records and speeds following neck cooling interventions at different temperatures. The values are expressed as mean ± SD. (**A**) Record; (**B**) speed; CON, control; LNC, low-temperature neck cooling; MNC, mixed-temperature neck cooling. * Significantly different versus first race (*p* < 0.05).

The changes in cycling race records and speeds following the three neck cooling interventions are shown in Figure 6. The two-way repeated-measures ANOVA conducted on race record (F = 23.472, *p* = 0.003) and speed (F = 61.649, *p* < 0.001) showed significant differences in time. In all trials, the cycling race record of the second race was significantly lower than that of the first race (CON, 147.57 ± 4.10 vs. 153.71 ± 5.60; LNC, 147.29 ± 4.95 vs. 149.43 ± 5.95; MNC, 147.86 ± 4.36 vs. 151.00 ± 3.66 sec, *p* < 0.05). Similarly, the speed of the second race was also significantly lower than that of the first race (CON, 47.42 ± 1.56 vs. 46.17 ± 1.50; LNC, 48.94 ± 1.65 vs. 48.26 ± 1.95; MNC, 48.83 ± 1.36 vs. 47.41 ± 0.85 km/h, *p* < 0.05). In addition, there was no significant difference between the trials (*p* > 0.05).

## 4. Discussion

The ability to sense and regulate body temperature is a key feature of human survival. A deviation of ±3.5 °C from resting temperature (37 °C) can lead to physiological impairments and fatality [17]. During exercise, metabolic heat production can increase by 10–20 times. When the heat-dissipating mechanisms cannot cope with metabolic heat production, body temperature increases [17]. Exercise duration and intensity that induce metabolic heat production significantly contribute to the amount of heat accumulated in the body [17,18]. The risk of heat injury due to exercise in cold environments is often underestimated. Several studies have reported that intense exercise can generate sufficient heat to cause injury, even in cold environments [17,19]. On the other hand, it has been suggested that cooling the neck during exercise in the heat can improve the performance of endurance athletes and the repeated sprint performance of team sport athletes [7]. Additionally, neck cooling during exercise has been reported to be a preferred cooling strategy for athletes over head and/or face cooling [7].

In this study, Tty and TS were measured and evaluated to verify the effect of neck cooling interventions at different temperatures on thermal stress in the body during cycling. Tty and TS significantly increased immediately after exercise in all trials. These results are believed to be primarily due to metabolic heat production during cycling. Meanwhile, TS significantly decreased 24 h after exercise compared to immediately after exercise in the LNC and MNC trials. Conversely, no significant difference was found in the CON trial. These results suggest that neck-cooling interventions may be effective in alleviating thermal perception. Several studies [20,21] have reported that neck cooling interventions can reduce the level of exercise-induced TS in a high-temperature environment, although it is not effective in reducing core body temperature. Bright et al. [20] reported that TS significantly reduced when a cooling collar was applied to 12 individuals while cycling for 90 min at an RPE of 16. Hamada et al. [21] reported that ice-pack cooling during bilateral carotid treatment can significantly reduce TS when cycling for 40 min at a VO_2_max of 60%.

An appropriate increase in the body and skeletal muscle temperatures promotes metabolic processes and facilitates exercise performance [22]. However, excessive increases in body temperature induced by intense exercise, environmental heat stress, or dehydration can result in significant cardiovascular strain [23]. In this study, HR and SpO_2_ were measured to verify the effects of neck cooling at different temperatures on physiological function during cycling. In all trials, HR significantly increased immediately after exercise, while SpO_2_ significantly decreased immediately after exercise. HR and SpO_2_ levels returned to pre-exercise levels 24 h after exercise. This suggests that the participants in this study performed two cycles of races to their maximum ability. Additionally, this suggests that the cardiovascular responses to cycling exercise returned to resting levels at 24 h after exercise. However, no significant differences were observed with the neck cooling intervention. This result supports previous studies [12,24,25] that reported no significant differences in HR and SpO_2_ with a neck cooling treatment. Tyler et al. [12] applied a cooling collar to healthy adult males during 75 min of running at 60% VO_2_max and 15 min of time-trial running. No significant difference in HR was reported after cooling under both exercise conditions. In a previous study by Torii et al. [24], an ice pack was bilaterally applied to the carotids for 20 min after 40 min of cycling. No significant difference in HR was observed compared to controls. Additionally, in a previous study in which cooling was applied to the head and neck of 12 healthy adults [25], no significant changes in resting HR and SpO_2_ were observed.

Previous studies [11,26] have suggested that cooling strategies such as CWI and cryotherapy can be effective in alleviating exercise-induced OS. Plasma or serum MDA concentrations reflect lipid peroxidation, and SOD—a representative antioxidant enzyme—has been reported as a biomarker for estimating the exercise-induced oxidant/antioxidant imbalance [27,28,29]. In this study, serum MDA levels and SOD activity were measured to verify the effects of neck cooling at different temperatures on oxidant/antioxidant status during cycling. In all trials, MDA and SOD levels significantly increased immediately after exercise. Subsequently, these levels decreased significantly 24 h after exercise, returning to pre-exercise levels. This is believed to be the result of cycling, causing OS to increase, and triggering concomitant antioxidant enzyme activities to increase as a defense mechanism. Cho et al. [29] reported a significant increase in blood MDA and SOD levels after acute exercise. This supports the results of the present study. However, no significant differences were observed between neck cooling interventions. These results suggest that the neck cooling treatment in this study did not have a significant effect on oxidant/antioxidant status, possibly because the cooling strategy did not induce a significant decrease in body temperature (especially core temperature). Hyperthermia has been suggested as a major cause of accelerated exercise-induced OS [30]. Previous studies have reported a decrease in OS after a significant decrease in body temperature [11]. Lindsay et al. [11] reported that CWI significantly reduced gastrointestinal temperature while alleviating exercise-induced inflammation and OS levels.

HSPs are molecular chaperones that facilitate the unfolding or folding of secondary structures of proteins and their target proteins under cellular stress conditions [31]. Various internal and external physiological and mechanical stresses cause homeostatic imbalances, resulting in increased HSP70 expression [31]. Exercise is also a stressor that can threaten protein homeostasis in various cell types. The increase in HSP70 after acute exercise reflects this mechanism [31]. In this study, we verified the effects of neck cooling at different temperatures on serum HSP70 levels during cycling. HSP70 levels increased significantly immediately after exercise in all trials. These results are consistent with those of previous studies that reported a significant increase in HSP70 levels after acute exercise [32,33]. It is believed that physiological stress—such as high body temperature and OS—induced by acute exercise increases HSP70 expression. Dalgaard et al. [32] reported that muscle HSP70 expression increased significantly after 4 h of cycling in 14 elite endurance athletes. In addition, Roh et al. [33] reported that serum HSP70 levels significantly increased following treadmill running at 75% of the heart rate reserve in 10 male college athletes. Meanwhile, the CON trial in this study showed a significant increase in HSP70 levels 24 h after exercise compared to immediately after exercise and showed significantly higher HSP70 levels than the LNC trial. These results suggest that the neck cooling interventions in this study did not have a significant effect on body temperature and OS. However, these interventions may be effective in reducing exercise-induced HSP70 expression. In a previous study, increased HSP70 expression was reported to be associated with exercise-induced muscle damage and inflammation [34]. Although in this study it could not be measured directly, it is thought that low-temperature neck cooling may be the result of reduced exercise-induced muscle damage. Pournot et al. [34] suggested that cooling treatment after exercise can alleviate exercise-induced muscle damage and inflammation. The low serum creatinine kinase level observed 24 h after exercise reflects this [34]. Future studies should verify this through an analysis of muscle damage biomarkers or inflammatory cytokines.

The physiological stress caused by exercise is related to hyperthermia, OS, muscle damage, inflammation, and nervous system fatigue. All of these conditions can reduce exercise performance [35]. In this study, cycling race records and speeds were measured to verify the effects of neck cooling interventions at different temperatures on exercise performance. The results of this study showed no significant differences between the different neck cooling interventions. These failed to alleviate physiological stress, such as body temperature, HR, and OS. A previous study [36] reported a significant improvement in exercise performance through neck cooling, accompanied by a significant decrease in core body temperature. In contrast, other studies [37,38] did not show significant differences in cycling performances with neck cooling. Similarly, no significant differences in core body temperature or HR were observed.

The above results suggest that, although neck cooling was not effective in improving exercise performance in adolescent cyclists, it may alleviate thermal perception and HSP70 expression in athletes during recovery after cycling training or competition. This study had several limitations. First, the participants in this study were adolescent cyclists from a single high school. Second, core body temperature, such as rectal temperature, and exercise-induced muscle damage and inflammatory response levels were not verified. Third, the participants in this study were all males. Therefore, this study could not account for all sexes. Finally, the study was conducted without exposure to environmental heat stress. Future studies should verify the effectiveness of cooling strategies under various environmental temperatures.

## 5. Conclusions

Carotid artery and cervical vertebrae cooling using a neck collar during cycling did not lead to a significant improvement in exercise performance or reduction in body temperature and OS in male adolescent cyclists. However, neck cooling may positively affect thermal perception during post-exercise recovery. Neck cooling at relatively low temperatures may be more effective in reducing exercise-induced HSP70 expression. Future studies should verify exercise-induced muscle damage biomarkers and inflammatory cytokines in a larger number of participants. Moreover, the usefulness of neck cooling should also be verified through long-term road cycling races under thermal-stress-inducing environmental conditions.

## Figures and Tables

**Table 1:** Physical characteristics of the participants (*n* = 7).

Characteristic	Mean ± SD	Range
Age (years)	17.00 ± 0.76	16.0–18.0
Height (cm)	174.16 ± 3.05	169.1–177.5
Weight (kg)	65.13 ± 2.12	63.1–68.7
BMI (kg/m^2^)	21.50 ± 0.73	20.1–22.1
SMM (kg)	31.79 ± 0.96	30.7–33.5
Fat Mass (kg)	8.91 ± 1.63	6.2–11.0
PBF (%)	13.61 ± 2.26	9.8–16.3
VO_2_max (mL/kg/min)	53.38 ± 1.34	52.1–55.8

BMI, body mass index; SMM, skeletal muscle mass; PBF, percentage of body fat; VO_2_max, maximal oxygen uptake.

## Data Availability

The data generated and analyzed during this study are included in this article. Additional data are available from the corresponding author on request.
